# Quantifying Poverty as a Driver of Ebola Transmission

**DOI:** 10.1371/journal.pntd.0004260

**Published:** 2015-12-31

**Authors:** Mosoka P. Fallah, Laura A. Skrip, Shai Gertler, Dan Yamin, Alison P. Galvani

**Affiliations:** 1 Community-Based Initiative, Ministry of Health, Monrovia, Liberia; 2 National Institute of Allergy and Infectious Diseases, PREVAIL-III Study, Monrovia, Liberia; 3 A.M. Dogliotti College of Medicine, University of Liberia, Monrovia, Liberia; 4 Center for Infectious Disease Modeling and Analysis, Yale School of Public Health, New Haven, Connecticut, United States of America; 5 Department of Industrial Engineering, Tel Aviv University, Tel Aviv, Israel; Liverpool School of Tropical Medicine, UNITED KINGDOM

## Abstract

**Background:**

Poverty has been implicated as a challenge in the control of the current Ebola outbreak in West Africa. Although disparities between affected countries have been appreciated, disparities within West African countries have not been investigated as drivers of Ebola transmission. To quantify the role that poverty plays in the transmission of Ebola, we analyzed heterogeneity of Ebola incidence and transmission factors among over 300 communities, categorized by socioeconomic status (SES), within Montserrado County, Liberia.

**Methodology/Principal Findings:**

We evaluated 4,437 Ebola cases reported between February 28, 2014 and December 1, 2014 for Montserrado County to determine SES-stratified temporal trends and drivers of Ebola transmission. A dataset including dates of symptom onset, hospitalization, and death, and specified community of residence was used to stratify cases into high, middle and low SES. Additionally, information about 9,129 contacts was provided for a subset of 1,585 traced individuals. To evaluate transmission within and across socioeconomic subpopulations, as well as over the trajectory of the outbreak, we analyzed these data with a time-dependent stochastic model. Cases in the most impoverished communities reported three more contacts on average than cases in high SES communities (p<0.001). Our transmission model shows that infected individuals from middle and low SES communities were associated with 1.5 (95% CI: 1.4–1.6) and 3.5 (95% CI: 3.1–3.9) times as many secondary cases as those from high SES communities, respectively. Furthermore, most of the spread of Ebola across Montserrado County originated from areas of lower SES.

**Conclusions/Significance:**

Individuals from areas of poverty were associated with high rates of transmission and spread of Ebola to other regions. Thus, Ebola could most effectively be prevented or contained if disease interventions were targeted to areas of extreme poverty and funding was dedicated to development projects that meet basic needs.

## Author Summary

Despite recognition that resource constraints contributed to the difficulty in controlling West Africa’s ongoing Ebola outbreak, no previous study has been conducted to quantify the impact of poverty on transmission. In particular, the extent to which within country heterogeneity in socioeconomic status (SES) could be driving geographic spread or sustaining Ebola hotspots has important implications for control policies. In Liberia’s capital city Monrovia, approximately 68% of the population occupies slum neighborhoods characterized by overcrowding and lacking sanitation—conditions conducive to outbreaks of infectious disease. The researchers find that infected individuals residing in these most impoverished settings are associated with three times more Ebola transmission, as well as greater dissemination of disease between communities of different socioeconomic levels. Cases from low and middle SES areas reported significantly more contacts during their symptomatic and therefore infectious periods than cases from high SES areas. Overcrowding and lack of education on how the disease is transmitted could explain the observed differences in number of secondary cases. The findings support the need for targeted response measures that account for differential risk arising from socioeconomic heterogeneity within West African countries.

## Introduction

The 2014–2015 Ebola outbreak continues to have a global impact. Prevention and preparedness measures remain in place at airports and hospitals across the United States and Europe, while ongoing transmission in West Africa has led to a case count exceeding 28,450 [1]. Since October 2014, however, the epidemic has been diminishing. Despite a recent resurgence in Liberia, efforts have shifted from emergency response to endgame strategies. Fundamental to preventing or mitigating future outbreaks is the identification of factors that exacerbate risks of Ebola emergence, transmission and geographical dissemination. While it has been appreciated that insufficient healthcare infrastructure in Liberia, Guinea and Sierra Leone has been a primary obstacle in the treatment of Ebola, socioeconomic heterogeneities within these countries have not been evaluated as determinants of Ebola transmission. In Liberia, specifically, more than 1.4 million of the country’s extreme poor have incomes less than $0.50 per day [2], adult literacy rates are under 43%, and public spending allocated to health is only 4% of the GDP [3]. Approximately, 68% of its urban population resides in a network of slums [4] characterized by overcrowding, high crime, and lack of sanitation [5–7]. Even for the remainder of the population, there are stark differences regarding population density and improved sanitation between middle and higher SES.

To determine the role of poverty in the Ebola outbreak, we analyzed transmission chains using a time-dependent stochastic model that was adapted to evaluate the heterogeneity of Ebola incidence and transmission factors for over 300 communities within Liberia between February and December 2014. We found that cases from low and middle SES regions have significantly more contacts when infectious and lead to much greater transmission than cases from higher SES regions. Nonetheless, all SES regions responded to instrumental interventions aimed at rapid hospitalization, concomitantly reducing community transmission and improving case fatality over the course of the outbreak.

## Methods

### Data

We analyzed data on Ebola incidence and case fatality for Montserrado County, Liberia provided by the Liberian Ministry of Health and Social Welfare (MoHSW) from two sources: 1) Case Classification Data (CCD) (S1 Data), and 2) Contact Tracing Data (CTD) (S2 Data). The CCD were collected using the Viral Hemorrhagic Fever form [8] and provide information about 4,373 individuals reported as suspected, probable, or confirmed Ebola cases in Montserrado County between February 28, 2014 and December 1, 2014. The CCD includes case classification status (i.e. suspected, probable, or confirmed), survivorship status, date of symptom onset, date of isolation when applicable, as well as general information regarding age, gender and community of residence. Upon removing duplicate entries and entries with no reported community, our dataset consisted of 3,532 individuals. The CTD provided additional information for a subset of 1,585 individuals who were traced between July 7, 2014 and October 28, 2014. The CTD specified the contacts encountered by each index case following symptom onset. Contacts were monitored for 21 days or until loss to follow-up or onset of symptoms. For contacts who became cases, tracing information was also recorded.

We classified 324 out of the 452 unique communities of residence that were identified from the CCD into three levels of SES (high, middle, and low) according to key indicators [9]. Communities were classified as high SES if residents tended to occupy modern/concrete structures; more than a third of households had access to improved sanitation; and population density was below the average for Monrovia (108,692 population per square kilometer [10]). Communities were classified as middle-to-low SES if most residents occupied tin roof homes; less than a third of households had access to improved sanitation; and population density was higher than the average for Monrovia. Out of the communities meeting these criteria, the subset consisting of slum neighborhoods, including West Point, New Kru Town, Clara Town/Struggle Community, Doe Community, People’s United Community, Logan Town, Jallah, Slipway, Peace Island, and Sinkor’s 12th Street, was classified as low SES. These low SES slums are characterized by high population density, elevated crime rates, ambiguous land ownership, unimproved water sources, and limited or no health care facilities, schools, and sanitation infrastructure [5–7]. Cases reporting areas of residence that were individual compounds or more general areas that consisted of multiple neighborhoods were not included in the classification. Population density was determined using data collected by the Community-Based Initiative, which mapped Monrovia’s neighborhoods as part of an active Ebola surveillance program.

### Data Analysis

The case classification dataset was analyzed to consider differences in key factors of transmission among cases categorized at low, middle or high SES levels. Descriptive statistics were calculated as means and standard deviations for continuous variables and frequencies and percentages for binary variables. Analyses of variance with ordered levels were used to assess differences in numbers of reported contacts and days between symptom onset and hospitalization. Chi-square tests were used to compare frequencies of care seeking and survivorship among the groups. P-values less than 0.05 were considered statistically significant.

### Model

#### Parameterization

We extended a model [11] that integrates Ebola disease progression of individual infections with contact data to quantify transmission within and among community types. The model was parameterized with daily viral load and daily contact rate of each infected individual to stochastically assign secondary cases. By integrating the CCD, we generated and incorporated a contact matrix for individuals across the 22 zones of Montserrado County (S1 Fig). Contact behavior was expected to change during the epidemic [12, 13], such as due to reduced movement between zones with increased use of blockades and curfews that were in flux and being established between July to September. We therefore generated separate matrices of contacts specifically for July, August and September, the months of greatest transmission and when contact behavior is expected to shift. Since the CTD only included cases through the first two weeks of October and since most interventions that would impact inter-zone movement or interactions had been instituted by the end of September, contacts in October and November were assumed to follow the same inter-zone matrix as those in September. Extending the previous model to include zone-specific contact structure enabled us to capture transmission patterns between communities.

A total of 2,500 confirmed and probable cases with symptom onset in July through November were included in the modeling analyses. Individuals reporting a potential source case in either the CCD or CTD were assumed to have been infected by their reported sources, while all other cases in the dataset were assigned probabilistically. The CCD were used to determine their date of symptom onset, time between onset and hospitalization if applicable, survivorship status, and location (*i*.*e*., hospital or community) of funeral if applicable. To address incomplete data, we generated a monthly distribution for each model parameter based on documented data and sampled values for infected individuals with missing information. All parameters and their distributions are detailed in Table 1.

**Table 1:** Distributions for model parameters.

Parameter	Distribution^1^	Description	Reference(s)
**Duration of incubation period (days)**	Survivors: Gamma(2.8640, 3.5058) Non-survivors: Gamma(2.5988, 3.3515)	Incubation period was assumed to be distributed according to a gamma distribution, the parameters of which were fitted to a subset of the Case Classification Data (CCD) for cases who identified the funeral of a case as the source of transmission. We restricted the distribution to between 1 and 21 days and also stratified for survivors and non-survivors.	Fitted to CCD data
**Overall duration of symptoms (days)**	Triangular(5,8,14)	A triangular distribution with mode of eight days was derived from published estimates.	[14–16]
**Duration of late symptoms**	Uniform(1,5)	The duration of the late symptoms phase duration was drawn from a uniform distribution ranging from one to five days, as has been clinically characterized by more severe symptoms including vomiting, diarrhea, hemorrhaging, and organ failure.	[14, 17]
**Daily viral load** ^**2**^ **per case**	Gamma (Varies per case)	Survivorship-specific daily viral load was sampled per person from a gamma distribution, fitted to published viral load data from the 2000–2001 Uganda outbreak.	[18]
**Rate ratio of transmission risk**	Gamma(4.5824, 0.5874) (S2 Fig)	Relative risk distributions for contact and survivorship-specific viral load were sampled using a Monte Carlo scheme to generate a distribution for the rate ratio. A gamma distribution was fit to the empirical distribution for viral load and truncated between 1 and 100.	[11, 18, 19]
**Number of contacts per epidemic month**	Raw data (S3 Fig)	A distribution was derived using available CCD and Contact Tracing Data (CTD) for each month. The range was truncated between 1 and 40 contacts.	Calculated from data
**Days from onset to hospitalization per epidemic month**	Raw data (S4 Fig)	A distribution was derived using available CCD for each epidemic month. The range was truncated at 20 days and sampled from only for cases reporting care-seeking but no date.	Calculated from data
**Days from onset to death per epidemic month**	Raw data (S5 Fig)	A distribution was derived using available CCD for each epidemic month. The range was truncated at 100 days.	Calculated from data
**Days from death to burial per epidemic month** ^**3**^	[3, 3, 1, 1, 0, 0]	A distribution was derived using available CCD for average number of days between death and date of funeral practices.	Calculated from data
**Probability of community burial after hospitalization per epidemic month** ^**3**^	[0.2727, 0.2697, 0.3306, 0.3883, 0.4293, 0.4150]	A distribution was derived using available CCD for non-survivors with reported care-seeking and for whom the burial location (i.e. hospital or community) was documented. Distributions were calculated for each epidemic month.	Calculated from data
**Probability of sanitary funeral per epidemic month** ^**3**^	[0, 0, 0, 0, 0.8, 0.8]	The probability of a sanitary burial given a community-based funeral.	[20]
**Zone contact matrix per epidemic month**	Raw data (Heat maps for contact matrices presented in S1 Fig)	Frequencies of inter- and intra-zone interactions were determined using the zones of residence of cases and their contacts reported in the CTD or CCD	Calculated from data

^1^ Distributions are presented as name of distribution followed by relevant parameters: Gamma(shape, scale), Triangular(lower bound, mode, upper bound), and Uniform(lower bound, upper bound).

^2^ Viral load was measured based on the mean and standard deviation counts of daily RNA copy levels following symptoms onset and are stratified by case fatality.

^3^ Data presented for each epidemic month and reported as [June, July, August, September, October, November].

#### Simulation study

To determine the likely source from which each case was infected, we conducted 1000 stochastic simulations of the model. We probabilistically sampled from the empirical distributions outlined in Table 1 to determine the relative likelihood of each individual in his or her infectious period to transmit to a newly infected individual over the course of an incubation period, given date of symptom onset. Cases who reported seeking care were assumed to no longer transmit within the community beyond their reported date of hospitalization. Given a month-specific probability of traditional burial, non-survivors could become a source case post-mortem. All deceased cases with hospital funerals were assumed to have hygienic burials and thus did not contribute to post-mortem transmission. The number of secondary cases was determined for each source from 1000 iterations of the model. In each simulation, we tracked the SES of sources and secondary cases to evaluate transmission patterns within and between community types. We then calculated a risk ratio by dividing the proportion of secondary cases in each SES group given the SES level of the source by the proportion expected under a homogenous model.

## Results

The case classification dataset was analyzed for the 3,532 cases meeting our inclusion criteria and with dates of symptom onset between February 28, 2014 and December 1, 2014. On average, relative to probable and confirmed cases of Ebola in high SES areas, cases in communities of middle and low SES reported seeking care less frequently, although the difference is not statistically significant (Table 2). For those who presented at an Ebola treatment unit (ETU) or other health care facility, there were no significant differences in time from symptom onset to hospitalization. A decreasing trend was observed in time to hospitalization for all three SES groups between July and November 2014 (S6 Fig).

**Table 2:** Key factors of Ebola transmission based on socioeconomic status (SES) of probable and confirmed cases^1^.

	High SES	Middle SES	Low SES	P-value^2^
	(n = 544)	(n = 1044)	(n = 456)	
Number of Contacts (mean ± SD)	7.41 ± 9.45	8.01 ± 8.53	10.31 ± 10.73	<0.001^3^
Time to isolation (mean ± SD)	4.58 ± 5.03	4.36 ± 3.15	5.33 ± 5.97	0.247
Hospitalization (n (%))	140 (33.98)	239 (27.86)	99 (27.89)	0.063
Mortality (n (%))	205 (42.53)	412 (41.70)	199 (46.50)	0.240

^1^ Continuous variables are presented in terms of mean and standard deviation per group; categorical variables are presented as number of individuals and percentage per group. Percentages are based on the number of individuals in the sample for whom care-seeking or mortality data were available.

^2^ P-values calculated using an analysis of variance with ordered levels for continuous variables and chi-square tests for count variables.

^3^ Statistically significant at the P = 0.05 level.

Cases in areas of low and middle SES were associated with a statistically significantly higher number of contacts. In particular, cases reporting residence in a low SES community had an average of nearly three more contacts, as compared to individuals residing in the high SES areas (10.31 versus 7.41 average contacts, p<0.001). Although the number of contacts reported by all SES groups increased over the course of the epidemic, cases from low SES communities were consistently associated with more (S6 Fig). No statistically significant differences in mortality rate were observed across the three SES levels (p = 0.240), but pairwise comparisons suggested that the case fatality for the low SES communities (46.50%) and middle SES communities (41.70%) tended to be higher than that (42.53%) for the high SES communities (p = 0.257 and p = 0.106, respectively).

Source cases reporting a low SES residence were associated with 3.5 times as many secondary cases as those sources reporting a high SES residence (95% CI: 3.1–3.9) (Table 3). In addition, the majority of index cases in the high SES areas transmitted to secondary cases in high SES areas, while only about a third of secondary cases originating from index cases in low SES areas were also in low SES areas. These observations provide evidence that cases tended to be exported from poverty areas and result in transmission chains across Montserrado County.

**Table 3:** Risk ratio of transmission to secondary cases in low, middle, or high SES communities, given SES of the source case.

		Secondary Cases			
		Low SES	Middle SES	High SES	Risk ratio
Source case	Low SES (17.2%)^1^	5.45 (5.05–5.87)^2^	1.79 (1.60–1.99)	1.26 (1.10–1.43)	2.16 (2.03, 2.31)
	Middle SES (35.4%)	0.65 (0.55–0.74)	1.13 (1.04–1.21)	0.91 (0.83–0.98)	0.94 (0.88, 0.99)
	High SES (47.4%)	0.31 (0.26–0.36)	0.56 (0.52–0.61)	0.78 (0.73–0.82)	0.62 (0.59, 0.66)

^1^ The percentage of the study population is provided for each SES category.

^2^ 95% confidence intervals are provided based on results from 1,000 stochastic simulations of the model.

## Discussion

Our analyses show that despite widespread poverty throughout Liberia, less developed, more resource-constrained areas in Montserrado County tended to have more contacts after Ebola symptom onset and lead to more widespread transmission than higher SES communities. The significantly higher number of reported contacts by sources from low SES areas is consistent with overcrowding and lack of education on routes of disease transmission and prevention. Overall, cases from middle and low SES communities were associated with 1.5 and 3.5 times as many secondary cases, respectively, as sources from high SES communities.

We found that infected individuals who were residents of low SES communities were more likely to export Ebola to other SES communities than infected individuals within higher SES communities. Thus, not only were low SES communities disease hotspots, but they were also catalysts of spread throughout Monrovia. Employment opportunities are typically outside of the lowest SES communities, leading to substantial daily movement back and forth from slums throughout Monrovia.

Our findings suggest that targeting areas of extreme poverty would have the greatest impact in terms of preventing or containing outbreaks of infectious disease. However, the construction and staffing of hospitals or other public health infrastructure in the most disadvantaged areas of Liberia is a daunting even if essential undertaking, particularly in the context of worsened socioeconomic conditions at the country level due to the recent Ebola outbreak [21]. Therefore, optimal resource allocation to prevent widespread infectious disease will require improvements beyond hospital infrastructure [22].

Poverty has been correlated with increased rates of other infectious disease. In malaria endemic settings, for instance, living in traditional or unimproved homes with mud walls, thatched roofs, and earth floors has been associated with over twice the risk of infection than living in modern homes [23]. In Liberia, more specifically, cholera and other diarrheal diseases are highly prevalent in densely populated coastal regions of Monrovia [24, 25]. Poor sanitation conditions in these urban slums, where less than 25% of households have access to improved facilities, along with a lack of trash removal, result in regular contamination of the high water table [25]. Furthermore, overcrowding provides an environment conducive to the rapid spread and challenging containment of infectious disease. Focusing on sustainable development in urban slums and other communities of low and middle SES could significantly reduce the risk of future infectious disease outbreaks.

Despite the lack of infrastructure in Liberia’s urban slums, strong social networks exist. These networks afford opportunities for grassroots efforts to effectively engage community members in combatting infectious disease. The potential of community-driven efforts to contain Ebola spread became apparent in September when culturally sensitive messaging was used with leaders in the West Point slum to facilitate active case finding. The effectiveness of this approach is evidenced by our finding of increasingly prompt hospitalization, reducing transmission within the community [26], as well as by the organized response to and control of the recent outbreak in Nedowein, Liberia. Thus although poverty substantially exacerbates Ebola transmission, the obstacles imposed by poverty are not insurmountable by a targeted approach tailored to the specific needs and challenges faced by impoverished communities.

## Supporting Information

S1 Data Case classification data. The dataset consists information about individuals reported as suspected, probable, or confirmed Ebola cases in Montserrado County between February 28, 2014 and December 1, 2014.
(XLSX)

S2 Data Contact tracing data. The dataset consists of information on 1,585 suspected, probable, or confirmed cases whose contacts were traced between July 7, 2014 and October 28, 2014.
(XLSX)

S1 Fig Heat maps depicting zone-level contact matrices for July, August, and September 2014. Montserrado County, Liberia is comprised of 22 zones. Case investigation data on the zones of residence for cases and their reported contacts were used to generate relative probabilities of contacts between and within zones. Increasingly less inter-zone contacts were observed from July to August to September. In the figure, the 22 zones are ordered as 1400, 1700, 100, 1900, 700, 300, 1600, 1300, 1500, 1000, 1200, 900, 800, 200, 600, 400, 1100A2, 1100B1, 1100B2, 1100A1, 500, 1800.
(DOCX)

S2 Fig Frequency distributions for the number of contacts per case from case investigation data. Data are provided per epidemic month in 2014.
(DOCX)

S3 Fig Frequency distributions for time between symptom onset and hospitalization from case investigation data. For each individual reporting current or prior hospitalization, the number of days between his/her date of symptom onset and date of hospital admission was calculated. Data are provided per epidemic month in 2014.
(DOCX)

S4 Fig Frequency distributions for time between symptom onset and death from case investigation data. For each non-surviving case, the number of days between his/her date of symptom onset and date of death was calculated. Data are provided per epidemic month in 2014.
(DOCX)

S5 Fig Histogram of a truncated gamma distribution for the rate ratio, or expected change in transmission rate given a tenfold change in viral load. (DOCX)

S6 Fig Temporal trends in Ebola-related behaviors and case fatality, stratified by socioeconomic status (SES). Data are provided per epidemic month in 2014.
(DOCX)
