# Supplementary figures and images for: Quantifying Poverty as a Driver of Ebola Transmission
Source: PLoS Negl Trop Dis. 2015 Dec 31;9(12):e0004260. doi: 10.1371/journal.pntd.0004260 (PMC4697799; doi:10.1371/journal.pntd.0004260)

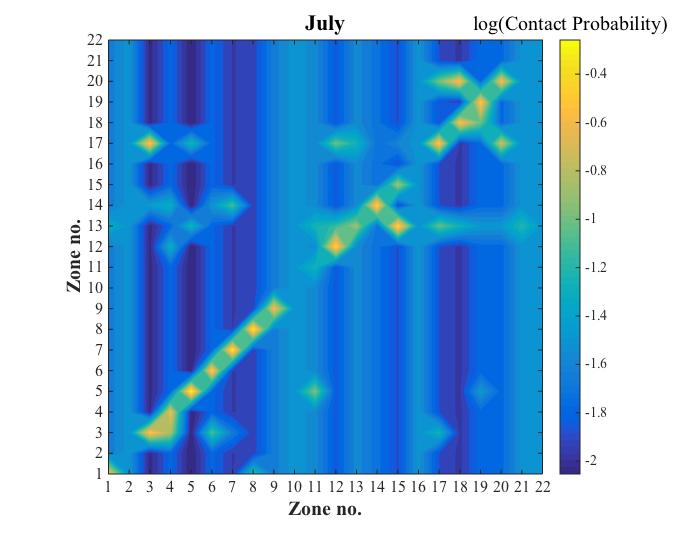

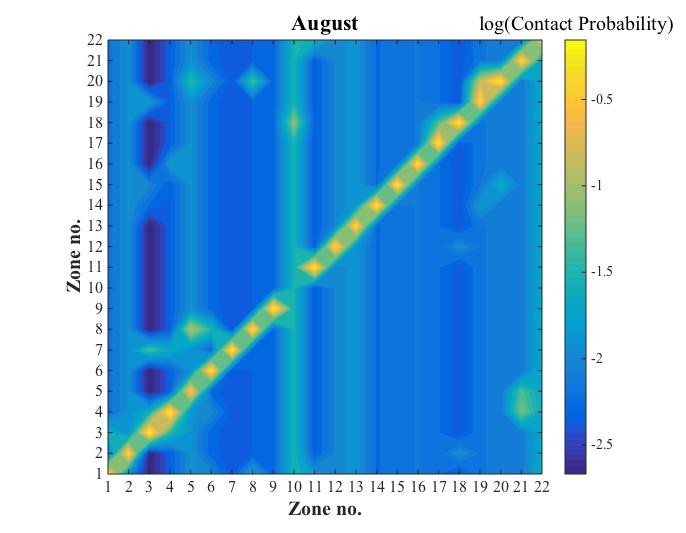

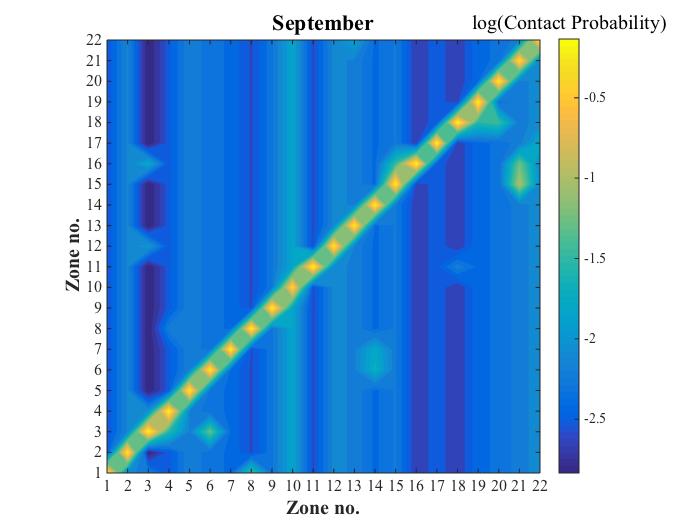

Supplement: S1 Fig — Montserrado County, Liberia is comprised of 22 zones. Case investigation data on the zones of residence for cases and their reported contacts were used to generate relative probabilities of contacts between and within zones. Increasingly less inter-zone contacts were observed from July to August to September. In the figure, the 22 zones are ordered as 1400, 1700, 100, 1900, 700, 300, 1600, 1300, 1500, 1000, 1200, 900, 800, 200, 600, 400, 1100A2, 1100B1, 1100B2, 1100A1, 500, 1800. (DOCX) [file pntd.0004260.s003.docx]

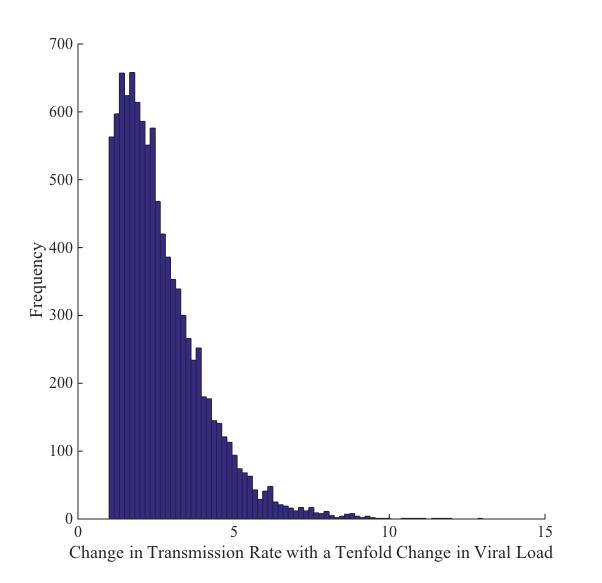

Supplement: S2 Fig — Data are provided per epidemic month in 2014. (DOCX) [file pntd.0004260.s004.docx]

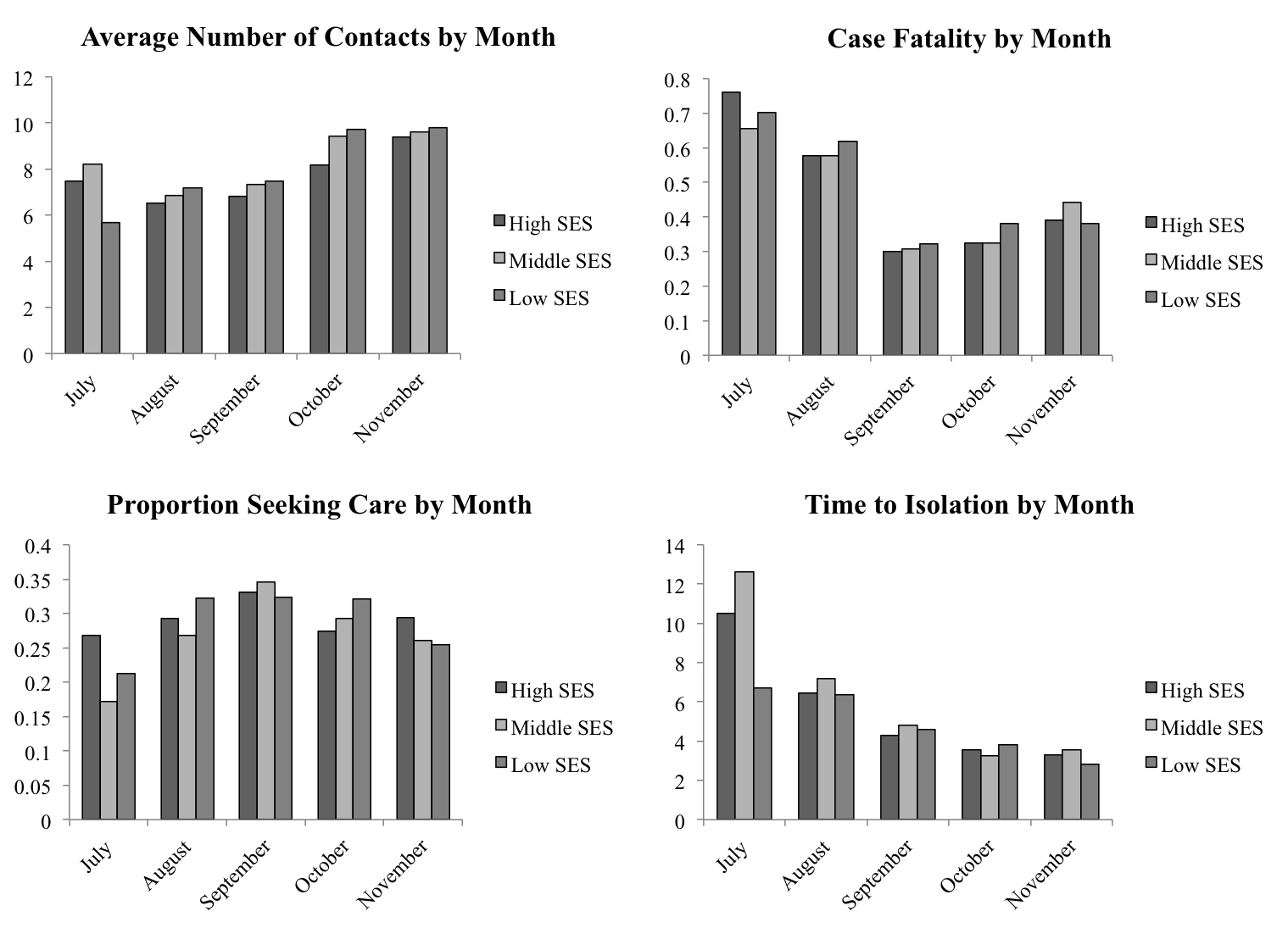

Supplement: S6 Fig — Data are provided per epidemic month in 2014. (DOCX) [file pntd.0004260.s008.docx]
