# Supplementary material for: Quantifying Poverty as a Driver of Ebola Transmission
Source: PLoS Negl Trop Dis. 2015 Dec 31;9(12):e0004260. doi: 10.1371/journal.pntd.0004260 (PMC4697799; doi:10.1371/journal.pntd.0004260)
Supplement: Supplementary file 6 [file pntd.0004260.s007.docx]

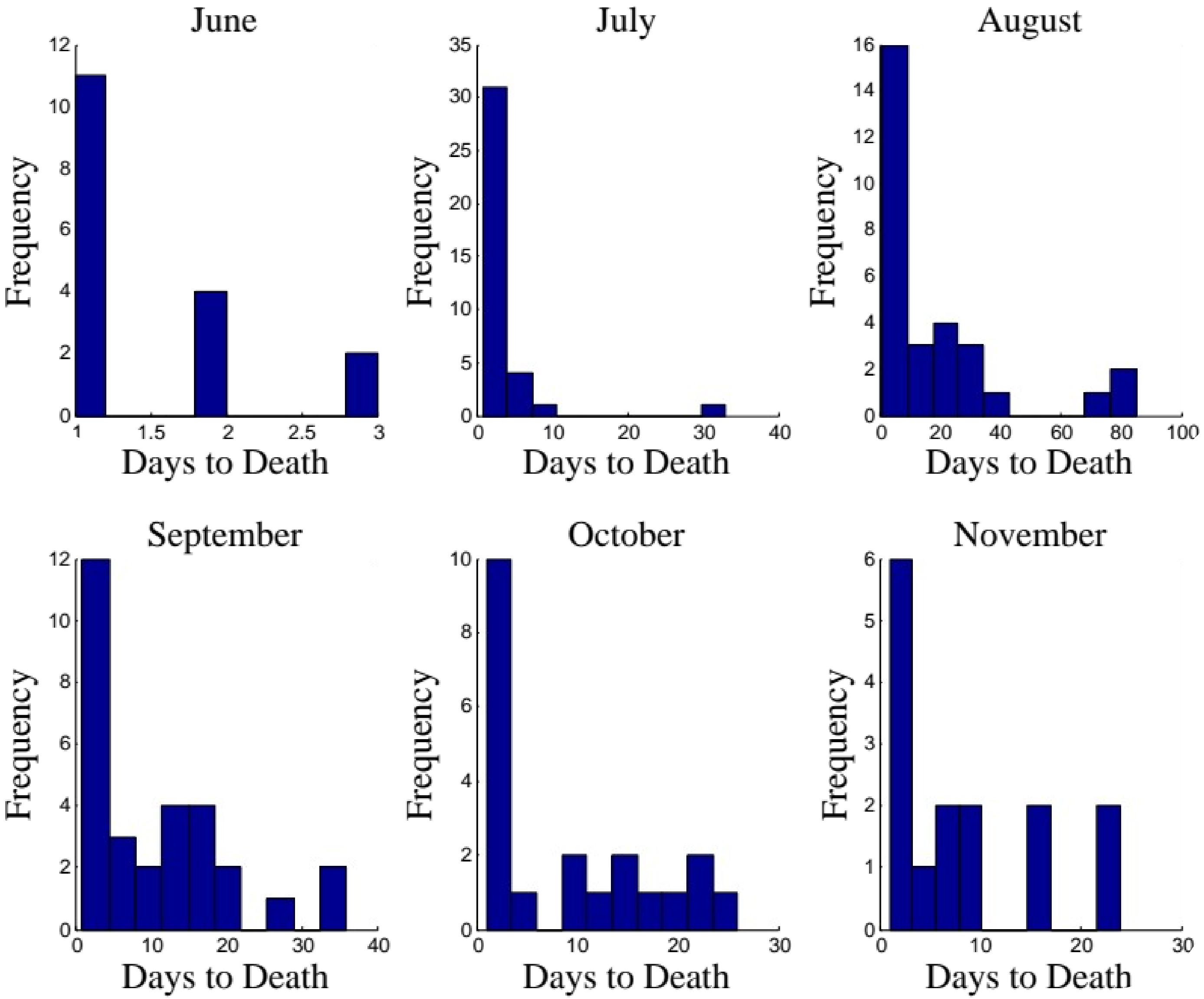

June
July
August
September
October
November
Frequency
Days to Death
